# Supplementary material for: Do alexithymia and negative affect predict poor sleep quality? The moderating role of interoceptive sensibility
Source: PLoS One. 2022 Oct 3;17(10):e0275359. doi: 10.1371/journal.pone.0275359 (PMC9529110; doi:10.1371/journal.pone.0275359)
Supplement: Attachment — Submitted filename: Response to Reviewers.pdf [file pone.0275359.s001.pdf]

Responses to Reviewers' comments:

Reviewer #1: ID: PONE-D-21-13329

Title: Do Alexithymia and Negative Affect Predict Poor Sleep Quality? The Moderating Role of Interoceptive Sensibility

Thank you for providing a chance to review this manuscript.

Comment:

Major revision.

Detailed information:

1. Introduction

1-1. Your introduction takes up nearly half of the entire article. Please write more concisely and distill your key points. Page 10-11 Line172-181: The purpose of the research in the introduction is very similar to the abstract. Please describe the corresponding theoretical support and hypotheses, and highlight the practical significance of your research. Page 10-11 Line176, "IS (MAIA)": This is really confusing.

Reply: We thank the reviewer for this important suggestion. We have shortened the Introduction section and edited the manuscript. Additionally, the terminology used in this article has been refined to enhance clarity. We have also revised the "Current Study" section and tried to provide a clear description according to your comment.

Line 145 to 158:

This study aimed to investigate the hyperarousal-related psychopathological mechanisms in sleep disturbances associated with alexithymia and negative affectivity in individuals with different IS characteristics. We hypothesized that, in individuals with a tendency to be internally focused and process bodily sensations emotionally (i.e., higher IS), physiological arousal may interfere with sleep quality in the expression of negative affect. In contrast, in individuals with a tendency to ignore or interpret bodily sensations non-emotionally (i.e., lower IS), physiological arousal may interfere with sleep quality in the expression of alexithymia. Thus, we used self-reported questionnaires to assess alexithymia, daily affective experiences, IS, and sleep quality among our community sample. Stepwise regression analysis of alexithymia, negative and positive affectivity on sleep quality in individuals with different IS characteristics was performed. We hypothesized that (1) a higher negative affect predicts worse sleep quality in individuals with higher IS tendency and (2) higher alexithymia predicts worse sleep quality in individuals with lower IS tendency.

2. Materials and Methods

2-1. Participants

Page 11 Line186-187, "3) normal vision (with or without correction)": Is this inclusion criterion necessary? And how do you manage the missing data?

Reply: We thank the reviewer for clarifying this issue. The data used in this study were part of a larger project. Although this criterion was not necessary for this study, data relevant to visual attention and color perception were collected for the entire project. Hence, this criterion was clearly stated in our online advertisements for project recruitment. Furthermore, all the participants acknowledged this requirement before they applied. Therefore, we did not actively exclude any individuals based on this criterion. For clarity in the manuscript, we have moved the description of the whole project from “Procedure” to “Participants” and added a further description for it.

Line 162 to 171:

The data are part of a larger project, the Human Project from Mind, Brain, and Learning (HPMBL) in Taiwan. HPMBL was approved by the Research Ethics Committee of Chengchi University, Taipei, Taiwan (NCCU-REC-201810-I074). The project concerns comprehensive well-being of adults in urban life. Online advertisements were posted on a university discussion webpage on social network media. The university is in a large city. The inclusion criteria were: 1) age 20 to 64; 2) native speakers of traditional Chinese; 3) normal vision (with or without correction) ; and 4) not diagnosed as having mental disorders that influence reality testing or cognitive ability, such as schizophrenia or dementia. The inclusion criteria were a combination to meet all the data collection requirement for the whole project.

2-2. Measurements

Page 11: You mentioned PSQI is a Chinese version, it’s a traditional one or simplified one? And how about the other measures? Did you use Chinese version too? If so, have they been validated? What’s the measurement properties of them? They should be concisely reported with details.

Reply: We thank the reviewer for this comment. All measures were in the traditional Chinese version. We have added relevant psychometric properties and Cronbach’s alpha in this study at the end of each measurement, lines 183, 197, 207, 229, and 238.

2-3. Procedure

Page 14: What is the response rate for obtaining data through online surveys? How do you control data quality?

Reply: We thank the reviewer for the clarification of this procedure. We apologize

that we did not clarify that all data were collected offline face-to-face. We have added a relevant description in the Procedure section.

Line 242 to 245:

The database project HPMBL was commenced in 2018. Multilevel constructs were collected using self-reported questionnaires and computerized tests. All the data collection was offline, such that questionnaires were all responded with paper and pencil.

3. Results

Page 20 Table 4: What is the meaning of R^2 if it’s too small? Furthermore, what is meaning of the regression model considering all R^2s are small?

Reply: We thank the reviewer for highlighting this critical issue. Sleep is a complex process; this study captured only a small part. We have added relevant discussion in the Discussion section, under the theme of limitations and future research.

Line 378 to 389:

Although the regression results in our study were statistically significant, the R^2s were all small, indicating a small effect of the studied variables. Sleep is a complex process influenced by the circadian, homeostatic, and arousal processes [2,74,75]. Emotion and alexithymia are only a part of the arousal process; hence, the contribution might in fact be small. Considering only the arousal process, several important variables were not included in this study. For example, hyperarousal was not assessed directly. We did not measure emotion regulation difficulty, which is partially embedded in the construct of alexithymia [13,14] and is intimately related to negative affective experience [32]. Self-reported sleep quality can be affected by interoception, which is a process relevant to homeostasis [76,77]. Incorporating these constructs could be informative for future studies.

4. Discussion

Page 21-22 Line 321-347: The “discussion” should be resented with the order of “results” are presented. What’s more, this part is a bit confusing, please be more organized and refined.

Reply: We thank the reviewer for this important suggestion. We have reorganized the discussion according to the order of results, and added the table references to the relevant sentence. Further, we modified the manuscript for improved logical flow, grammar, language, and clarity.

5. Taking all the comments above into consideration, this paper is interesting and written with a lot of work, but some issues may still need to be reconsidered. I hope you can further adjust the construct of your content. And it is of great importance to request a native English speaker to check your writing and make the expression more understandable.

Reply: We thank the reviewers for their encouraging and thoughtful comments. After completing these adjustments, the manuscript was edited by a professional and native English speaker. We hope that our efforts improve the quality of this manuscript accordingly.

My bests.
Your reviewer.

Reviewer #2:

The current study submitted to PLOS One aimed to investigate hyperarousal-related psychopathological mechanisms in sleep disturbances associated with alexithymia and negative affectivity, as well as the possible moderating role of interoceptive sensibility in a population-based sample. The study found that interoceptive sensibility tendency moderates the psychopathological mechanism of poor sleep quality. Further, the study concluded that the group differences in alexithymia and positive affect provided further insight into emotional processing based on previous theoretical considerations.

In general, the study gives a thorough scientific background on the subject and uses rigid subjective methods with many standardized scales (PSQI; TAS; MAIA; PANAS and more) to explore the relationship.

This study adds a small contribution to the existing literature on sleep and different emotional and behavior traits; however, a few points should be addressed before publication.

Reply: We thank the reviewer for the encouraging and productive comments. We have tried our best to revise the manuscript based on your suggestions.

1. Abstract

The number of females participants is around 70% and instead of stating that in numbers in the methods section (abstract), it would be beneficial to the reader to see the percentage.

Reply: We thank the reviewer for this comment. We have changed the expression in line 37: A sample of 224 (70.1% were female) participants…

2. Introduction

The introduction gives a thorough review covering previous work on topics such as hyperarousal, emotions, alexithymia, interoceptive sensibility, and how they intertwine with sleep quality, insomnia, and overall sleep disturbances.

It struck me that just the introduction is roughly 2000 words (8 pages) and 80 references. Scientific papers usually tend to aim for 500–1000 words in the introduction and I highly recommend shortening the introduction (possibly move to methods, discussion, and cut down on the word count). Further, I would recommend limiting the excess number of references.

Reply: We thank the reviewer for this important suggestion. We have shortened the Introduction section and removed some issues that were not necessary for this study. The references have been decreased according to the reduction in the Introduction section. Additionally, the manuscript was edited by a professional and native English

speaker.

3. Materials and Methods

3-1. It would be of use to state where participants were recruited from? Was the online advertisement in a large city? University setting? Urban or rural area?

Reply: We thank the reviewer for clarifying this issue. This study was conducted in a university setting in a large city. Therefore, we have added a relevant description in Participants section.

Line 162 to 167:

The data are part of a larger project, the Human Project from Mind, Brain, and Learning (HPMBL) in Taiwan. HPMBL was approved by the Research Ethics Committee of Chengchi University, Taipei, Taiwan (NCCU-REC-201810-I074). The project concerns comprehensive well-being of adults in urban life. Online advertisements were posted on a university discussion webpage on social network media. The university is in a large city.

3-2. It would be beneficial to report what software the statistical analysis were run in and add the information to the Statistical Analysis section.

Reply: We thank the reviewer for clarifying this issue. The statistical analyses were conducted using SPSS Windows software version 21. This information has been added to lines 262–263.

4. Results

4-1. In my opinion the lack of participants characteristic table is a problem. The authors jump straight into Partial Correlation of Measures (Table 1) but there is missing information regarding participants characteristics, score on the scales and possible some background information on the participants (age, height, social status) if that was collected in the study.

Reply: We thank the reviewer for clarifying this issue. Based on your suggestion, we have expanded the first section of the Results into “Sample characteristics and partial correlation of measures.” The scores on the scales for the three interoception sensibility groups were originally presented in Table 3 in the early version of the manuscript. An “All Participants” column to the table and moved it to Table 1.

Line 266 to 269:

Sample characteristics and partial correlation of measures

The sample characteristics and scores of measures are presented in Table 1. The

partial correlation of all measures controlling for social desirability are presented in Table 2.

line 274:

Table 1. Sample Characteristics and Differences of Measures between Three IS Clusters.

4-2. Further, a short description on the gender difference and how they performed on the test would also give the reader a better insight into the results. For example: did females report more alexithymia? Where there any gender differences regarding Pittsburgh Sleep Quality Index scores?

Reply: We thank the reviewer for highlighting this critical issue. We tested for gender differences by controlling for response bias. We did not find gender differences in the total score on any scale. Only three subscales differed. We added a brief description in the “Sample characteristics and partial correlation of measures” section of the Results.

Line 269 to 272:

We also tested the gender differences on all measures controlling for social desirability. Males are higher on the MAIA-Attention Regulation (F = 4.05, p = .045), MAIA-Self-Regulation (F = 4.39, p = .037), and TAS-EOT (F = 6.37, p = .012). All other measures were not significantly different from male to female.

5. Discussion

The reviewer would like a short sentence regarding the possible self-selection bias which is always a risk when studies use general population samples chosen via advertisement.

Further a discussion is needed regarding the gender split of the participants. Females where 70% participants and previous research has shown that there is gender difference in both sleep behavior and mental aspect such as reporting of anxiety, alexithymia and depression.

Reply: We thank the reviewer for pointing out these two essential issues. According to your suggestion, we have analyzed gender differences and found no apparent differences. We propose that this is relevant to self-selection bias. The high homogeneity of participants may “override” gender differences. Hence, we have added a short discussion in the last paragraph of the Discussion section to address these limitations.

Line 371 to 378:

Future research should address these limitations. The participants were community samples recruited through online advertisements. Hence, self-selection bias was unavoidable. A higher homogeneity of participants may also lead to other problems in addition to a lack of generalizability. We did not find significant gender differences in self-reported negative affectivity, alexithymia, or sleep quality, as in previous studies [71-73]. This may be due to the high homogeneity that overrides gender differences. A study sample with a more comprehensive profile or clinical participants would increase the study's ecological validity.

6. Other comments

6.1 Line 63 – instead of saying "some studies" it can be written as other studies in order to prevent repetitiveness (from line 61 and line 65).

Reply: We thank the reviewer for this comment. The entire Introduction section has been shortened and proofread by an English professional editor; thus, the relevant expression has been modified as follows:

Line 89 to 92

Some evidence has shown higher levels of arousal among high-alexithymia individuals under neutral or baseline conditions [15-17] and in the recovery phase [18]. This persistent tonic hyperarousal may further affect sleep quality.

6.2 Line 343 – Recommend using effective instead of affective in the sentence: "somatic hyperarousal may be experienced as an affective way"

Reply: Thank you for your valuable comment. We have revised this sentence according to your suggestion in line 355–356.

6.3 Line 371 – there is NA ., and than a large letter after the , that should to be fixed.

Reply: We have revised this sentence according to your suggestion. Thank you.

Line 326 to 327:

Further, TAS was more strongly correlated with PA than NA (Table 2). Given the high emphasis on emotion regulation in the case of alexithymia,
