# Supplementary material for: Do alexithymia and negative affect predict poor sleep quality? The moderating role of interoceptive sensibility
Source: PLoS One. 2022 Oct 3;17(10):e0275359. doi: 10.1371/journal.pone.0275359 (PMC9529110; doi:10.1371/journal.pone.0275359)
Supplement: Attachment — Submitted filename: Response_to_Reviewers.docx [file pone.0275359.s002.docx]

**Responses to Reviewers**

Reviewer 1 ID: PONE-D-21-13329 Title: Do Alexithymia and Negative Affect Predict Poor Sleep Quality? The Moderating Role of Interoceptive Sensibility. I appreciate your efforts to improve the manuscript and to respond to the comments made in the first review process. However, there are still minor issues that need to be addressed.

Comment: minor revision.

Detailed information:

- **Abstract**

1. This section is still too long, especially the "Objectives" part, please simplify it appropriately.

**REPLY**: We thank the reviewer for this suggestion. We have shortened the objective and conclusions. The abstract are 218 words after revision.

**Line 27-55**

## Objectives

Emotion-related hyperarousal is an important core pathology of poor sleep. Studies investigating the interplay of alexithymia and affective experiences in determining sleep quality have yielded mixed results. To disentangle the inconsistency, this study examined the concurrent predictive power of alexithymia, and negative and positive affect, while incorporating interoceptive sensibility (IS) as a possible moderator.

## Methods

A sample of 224 (70.1% were female) participants completed the Toronto Alexithymia Scale, Positive and Negative Affect Schedule, Pittsburgh Sleep Quality Index, Multidimensional Assessment of Interoceptive Awareness (MAIA), and Marlowe‐Crowne Social Desirability Scale (for controlling response bias) using paper and pencil. A two-stage cluster analysis of the MAIA was used to capture IS characteristics. Stepwise regression was conducted separately for each IS cluster.

## Results

A three-group structure for IS characteristics was found. Higher alexithymia was predictive of poor sleep quality in the low IS group, while higher negative affect predicted poor sleep quality in the moderate and high IS groups. Additionally, alexithymia and positive affect were significantly different in the three IS groups, while negative affect and sleep quality were not.

## Conclusions

Emotion and cognitive arousal may impact sleep quality differently in individuals with different levels of internal focusing ability, depending on physiological versus emotional self-conceptualization. The implications on pathological research, clinical intervention, study limitations and future directions are discussed.

- **Materials and methods Participants**

1. Line 167, page 7, “The university is in a large city”: This means nothing to me and the readers. I suggest removing it.

**REPLY**: The authors thank reviewer for this suggestion. We have removed it.

2. Line 173 – 174, page 8: All number digits should be unified. Check other places too. This section is not very fluent and seems like several different parts spliced together. I would suggest combining and trimming some of the sentences to make them easier to read.

**REPLY**: The authors thank reviewer for this suggestion. We have checked the number digits through the manuscript, and revised this section as following:

**Line 160-171**

The data are part of a larger project, the Human Project from Mind, Brain, and Learning (HPMBL) in Taiwan. HPMBL concerns comprehensive well-being of adults in urban life, and was approved by the Research Ethics Committee of Chengchi University (NCCU-REC-201810-I074). Online advertisements were posted on a university discussion webpage on social network media. The inclusion criteria for HPMBL were: 1) age 20 to 64; 2) native speakers of traditional Chinese; 3) normal vision (with or without correction); and 4) not diagnosed as having mental disorders that influence reality testing or cognitive ability, such as schizophrenia or dementia. A total of 249 participants participate the HPMBL; 25 participants had missing questionnaire data for this study. Hence, a final sample of 224 participants was included in this analysis. The mean age of final sample was 22.13 years (SD = 2.71), and there were 157 (70.10%) women and 67 (29.90%) men.

3. Measurements Line 185-186, page 8; line 199, page 9; line 239, page 11: The reliability of this study should be placed in the "Results" section.

**REPLY**: The authors thank the reviewer for this suggestion. We have moved reliability of this study into the Results and renamed the first section as “Characteristics of measures and three IS clusters.”

**Line 260-262**

The overall Cronbach’s alpha of PSQI was 0.89; 0.74 for TAS; 0.84 for PA and 0.90 for NA; 0.91 for MAIA; 0.68 for Marlowe-Crowne Social Desirability Scale.

4. Procedure Line 250-253, page 11: "NTD" or "NTDs"? Please express it uniformly.

**REPLY**: We are sorry for this error; it was unified as NTD after revision.

- **Results**

1. Line 267 – 269, page 12: Please state vital results in the tables and change these two sentences with "(Table 1)" and "(Table 2)", which may make the expression clearer.

**REPLY**: The authors thank the reviewer for the suggestions. We have revised these two sentences. In addition, we noticed that in our previous revision, we added information according to three IS groups in Table 1, however mention our clustering in Table 3. Hence, we also restructured the Results and tables. Now, the Table 1 presents how the three group of IS constructed. The Table 2 shows the demographic and measure attributes of participants. The Table 3 presents correlations between all measures.

**Line 262-276**

The overall Cronbach’s alpha of PSQI was 0.89; 0.74 for TAS; 0.84 for PANAS-PA and 0.90 for PANAS-NA; 0.91 for MAIA; 0.68 for Marlowe-Crowne Social Desirability Scale. According to the final centroids (Table 1), the three IS clusters were categorized as low, moderate, and high IS groups. There was no significant difference of demographic data between the three groups. TAS and PANAS-PA scores showed statistically significant difference, such that the high IS group exhibited lower TAS and higher PANAS-PA; PANAS-NA and PSQI were not significantly different among the groups (Table 2). We also tested the gender differences on all measures controlling for social desirability. Males are higher on the MAIA-Attention Regulation (*F* = 4.05, *p* = .045), MAIA-Self-Regulation (*F* = 4.39, *p* = .037), and TAS-EOT (*F* = 6.37, *p* = .012). All other measures were not significantly different from male to female.

After controlling for social desirability, except for the two reversed scales (N-D and N-W), the MAIA subscales were profoundly related to each other. TAS was associate with PANAS-PA and PANAS-NA; in contrast, PANAS-PA and PANAS-NA was not associated. PSQI was significantly associated with TAS, PANAS-PA, and PANAS-NA (Table 3).

2. Line 270 – 271, page 12: Please standardize the number of decimals appearing in the whole text. Table 1 – Table 4: 1) All scientifical tables should be formatted as three-line tables. 2) All abbreviations (e.g., "IS" in Table 1) appearing in the table should be written in full at the bottom note of the table.

**REPLY:** The authors thank the reviewer for this remind. We have standardized the number of decimals as two (except for *p* values), and added “0” before decimal points when applicable. The formatting and footnote of tables were also adjusted.

- **Discussion**

1. Line 371 – 389, pages 23 – 24: 1) "Limitations" discusses the shortcomings of the study itself, while "future directions" is not only to improve the shortcomings of the study but also to write about the aspects of in-depth research. Also, you should more clearly list the strengths of this study. Understanding the difference between the two, I believe you can write this section in a more logical way. I suggest dividing your opinions into “strengths and limitations” and “future directions” with subtitles and paragraphs.

**REPLY:** The authors thank the reviewer and we have revised the discussion part per your important suggestion. The section “strengths and limitations” was revised, and a future direction section was added.

**Line 369-401**

There are several strengths and limitations of this study. By incorporating IS, this study raised fresh insights on psychopathology and interventions for sleep disturbances. The individual difference of IS provide a possibility to explain the inconsistent findings of alexithymia, negative affect and sleep problem. Accordingly, individualized intervention for sleep problems can be designed. Apart from the main focus of the study, we have also found a potential differentiation between PA and NA in their association strength with somatic feelings. The participants were community samples recruited through online advertisements. Hence, self-selection bias was unavoidable. A higher homogeneity of participants may also lead to other problems in addition to a lack of generalizability. We did not find significant gender differences in self-reported negative affectivity, alexithymia, or sleep quality, as in previous studies [71-73]. This may be due to the high homogeneity that overrides gender differences. In sum, this study’s ecological validity is not ideal. Although the regression results in our study were statistically significant, the *R*^2^s were all small, indicating a small effect of the studied variables. Sleep is a complex process influenced by the circadian, homeostatic, and arousal processes [2,74,75]. Emotion and alexithymia are only a part of the arousal process; hence, the contribution might in fact be small.

Several directions can be applied in future studies. First, IS is a subcomponent of interoception, the “sensing the physiological condition of the body, as well as the representation of the internal state within the context of ongoing activities, and is closely associated with motivated action to homeostatically regulate the internal state” [76, p.693]. Other domains of interoception can be investigated. Similarly, there are multiple levels and measures of affect and sleep quality. Incorporating different levels of such construct, especially physiological assessments of interoception (e.g., brain imaging of insula, heart-evoked potential heartbeat detection task), affect (e.g., brain and autonomic reactions of emotion induction), and sleep (e.g., polysomnography) can provide important information. Second, emotion regulation is an important factor involved with arousal-related sleep problem intervention. Additionally, emotion regulation is embedded in the construct of alexithymia [13,14], intimately related to negative affective experience [32], and highly relevant to interoceptive awareness [36]. Future study may incorporate emotion regulation in addition to the constructs of this study. Third, sleep and interoception may interplay within aspects other than hyperarousal. For example, interoception is also directly linked to homeostasis [76], another determined process of sleep quality [77]. Some possibilities of the relations between sleep and interoception have been discussed, however more empirical studies are required to determine such interplay [78]. Beyond the relationship between sleep and interoception, our finding further suggests that individual differences of interoception is associated with the express of differential mechanism of sleep process. This phenomenon can be examined in homeostatic relevant aspects of sleep.

2. 2) The “conclusion” part of the study is missing. This draft is a proper improvement, and I believe you have put a lot of effort into it. I hope you can make further revisions to increase the readability and standardization of the article. Thank you and my best, Your reviewer

**REPLY:** The authors thank the reviewer for the important suggestions and encouragement. A conclusion part was added per your suggestion.

**SLine 410-416**

**Conclusion**

We tested a novel idea that how an individual process their own bodily sensations associates with sleep disturbances using self-reported questionnaires. Alexithymia predicts lower sleep quality among low IS group. In contrast, negative affectivity predicts lower sleep quality among moderate and high IS groups. Further examination of psychopathology and intervention of sleep disturbance incorporating IS should be implemented in future.
